# Proposed three-phenylalanine motif involved in magnetoreception signalling of an Actinopterygii protein expressed in mammalian cells

**DOI:** 10.1098/rsob.230019

**Published:** 2023-11-22

**Authors:** Brianna Ricker, Sunayana Mitra, E. Alejandro Castellanos, Connor J. Grady, Daniel Woldring, Galit Pelled, Assaf A. Gilad

**Affiliations:** ^1^ Department of Chemical Engineering and Materials Sciences, Michigan State University, East Lansing, MI, USA; ^2^ Department of Biomedical Engineering, Michigan State University, East Lansing, MI, USA; ^3^ Department of Radiology, Michigan State University, East Lansing, MI, USA; ^4^ Department of Mechanical Engineering, Michigan State University, East Lansing, MI, USA

**Keywords:** glycosylphosphatidylinositol (GPI) anchor, magnetoreception, HaloTag, *Kryptopterus vitreolus*, GCaMP6m, phosphatidylinositol-specific phospholipase C (PI-PLC)

## Abstract

Studies at the cellular and molecular level of magnetoreception—sensing and responding to magnetic fields—are a relatively new research area. It appears that different mechanisms of magnetoreception in animals evolved from different origins, and, therefore, many questions about its mechanisms remain left open. Here we present new information regarding the Electromagnetic Perceptive Gene (EPG) from *Kryptopterus vitreolus* that may serve as part of the foundation to understanding and applying magnetoreception. Using HaloTag coupled with fluorescent ligands and phosphatidylinositol specific phospholipase C we show that EPG is associated with the membrane via glycosylphosphatidylinositol anchor. EPG's function of increasing intracellular calcium was also used to generate an assay using GCaMP6m to observe the function of EPG and to compare its function with that of homologous proteins. It was also revealed that EPG relies on a motif of three phenylalanine residues to function—stably swapping these residues using site directed mutagenesis resulted in a loss of function in EPG. This information not only expands upon our current understanding of magnetoreception but may provide a foundation and template to continue characterizing and discovering more within the emerging field.

## Graphical abstract

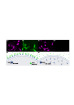

## 1. Introduction

In recent years, several organisms from all walks of life have been proposed to have magnetoreceptive properties. Magnetotactic bacteria use a membrane-bound iron-containing crystal, known as the magnetosome, to localize and move in relation to the Earth's magnetic field [1]. Migratory birds have been proposed to use cryptochromes (Cry4) located in the eye, or magnetite-based receptors in the beak to navigate the globe using its inherent magnetic field [2]. Recent studies have also demonstrated the human brain to possess magnetoreceptive properties [3]. The ability to sense and respond to magnetic fields is also well documented in diverse groups of fishes [4]. Marine animals including but not limited to medaka and zebra fish [5], glass catfish [6], eel [7], and sea turtles [8] have been proposed to sense magnetic fields for diverse purposes such as navigation, predator evasion or ontogenesis [9].

Despite the volume of research dedicated to magnetoreception, its exact mechanism remains elusive—especially that which is present in marine life [10]. In an effort to learn more about magnetoreception, RNA from the ampullary organ of the magnetoreceptive glass catfish (*Kryptopterus vitreolus*) was analysed, leading to the discovery of the novel Electromagnetic Perceptive Gene (EPG) in 2018 [11]. Previous studies on EPG indicated that it can be expressed in mammalian cells, and that its function is indicated by an increase in intracellular calcium upon stimulation with an electromagnetic field (EMF) [11,12]. Further studies explored the potential for EPG to be used to remotely treat nervous system disorders [13], and as a method of remotely controlling the activation of synthetic circuits [14]. These studies successfully confirmed that remote activation of EPG is possible *in vitro* and *in vivo*, but express the need for EPG to be structurally and functionally characterized in order to optimize its function for the system at hand.

In this paper, we aimed to elucidate information about the structure, function, localization, and molecular signalling pathway of EPG expressed in mammalian cells. HaloTag proved to be useful throughout this study as a tag that forms strong covalent bonds with various substrates that serve many diverse purposes [15]. HaloTag and its fluorescent ligands were used for imaging purposes that allowed for simple elucidation of EPG's localization. The fluorescent ligands also allowed for specific visualization of proteins of interest in cell lysate run on SDS-PAGE gels.

We report for the first time that EPG is present in the extracellular space and is associated with the membrane via a glycosylphosphatidylinositol (GPI) anchor. We also determined that a region rich in phenylalanine residues—unique to EPG—is critical for its function by observing calcium changes in our devised functional assay. Overall, this study provides important pieces of information about EPG's structure and function that lead us closer to using magnetoreception as an efficient synthetic tool as well as understanding magnetoreception at the cellular and molecular level.

## 2. Results and discussion

### 2.1. EPG is a membrane associated protein with its N terminus located extracellularly

It has been previously reported that EPG is associated with the plasma membrane in HEK293 cells [12]. The next meaningful question to answer is what orientation EPG takes in relation to the membrane. To that purpose, two vectors were created that fuse EPG to HaloTag. Halo-N-EPG consists of HaloTag fused to the N terminus of EPG; preceding HaloTag is EPG's known signal sequence [11]. Halo-C-EPG consists of HaloTag fused to the C terminus of EPG. These HaloTag-EPG fusion proteins allow for the use of selectively permeable HaloTag ligands to determine the membranal orientation of EPG. One fluorescent ligand used, Janelia Fluor X 650 (JFX650), is permeable to the cell membrane. The other fluorescent ligand used, Alexa Fluor 488 (AF488), is impermeable to the cell membrane.

**Figure 1.**
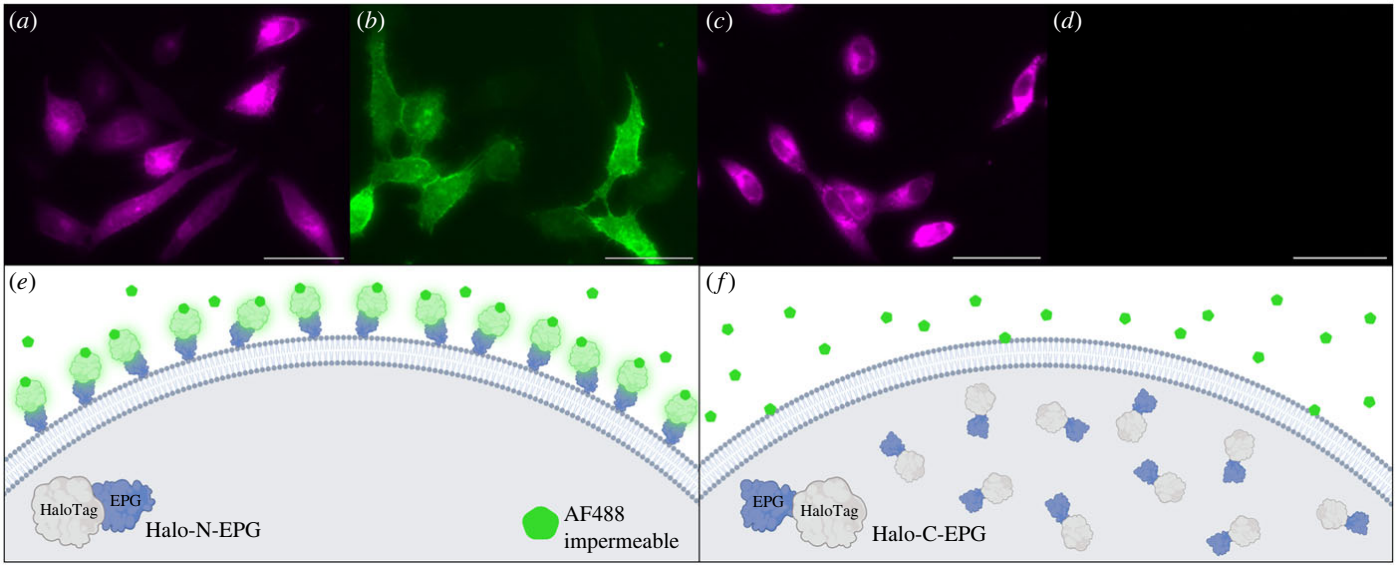
Fluorescently labelled HaloTag indicates EPG's cellular localization. (*a–d*) HeLa cells expressing Halo-N-EPG (*a,b*) and Halo-C-EPG (*c,d*) labelled with fluorescent HaloTag ligands: (*a,c*) membrane permeable JFX650, and (*b,d*) membrane impermeable AF488. (*e*,*f*) Illustrations to demonstrate the interaction of HaloTag-EPG fusion proteins with AF488. (*e*) As reflected in (*b*), Halo-N-EPG expressing cells were labelled with AF488 indicating its presence as a membrane associated protein with its N terminus exposed to the extracellular space. (*f*) As reflected in (*d*), Halo-C-EPG expressing cells were not labelled with AF488, indicating this construct does not localize to the membrane. Scale bars represent 50 µm.

HeLa cells expressing Halo-N-EPG were labelled with JFX650 (figure 1*a*) and AF488 (figure 1*b*). Binding of JFX650 indicates adequate expression of Halo-N-EPG by the cells, while binding of AF488 indicates that, minimally, the N terminus of EPG is exposed to the extracellular space. HeLa cells expressing Halo-C-EPG were labelled with JFX650 (figure 1*c*) and AF488 (figure 1*d*). Binding of JFX650 indicates adequate expression of Halo-C-EPG by the cells, and the lack of binding of AF488 indicates, minimally, that HaloTag (and the associated C terminus of EPG) is not exposed to the extracellular space. Together, these data indicate that EPG is associated with the plasma membrane with its N terminus facing the extracellular space.

### 2.2. Evidence that EPG is associated with the membrane via glycosylphosphatidylinositol anchoring

**Figure 2.**
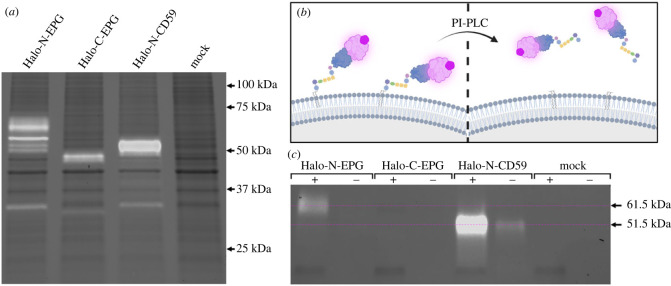
Lysate analysis and PI-PLC digestion signify EPG undergoes posttranslational modification to receive a glycosylphosphatidylinositol anchor. (*a*) Lysate of HeLa cells expressing HaloTag fusion proteins labelled with JFX650 run on an SDS-PAGE gel visualized with Far-Red excitation and 715/30 filter emission (white bands), and Stain-Free imaging (black bands). The expected size of the HaloTag fusion proteins is approximately 46 kDa. Halo-N-EPG presents as a series of bands between 50 and 75 kDa. Halo-C-EPG exhibits a band at 46 kDa. Halo-N-CD59 presents a band just above 50 kDa. Mock transfected cells do not present JFX650 associated bands. (*b*,*c*) HeLa cells expressing HaloTag fusion proteins labelled with JFX650 were treated with PI-PLC, effectively releasing any GPI anchored protein from the membrane as illustrated by (*b*). Media from treated (+) and untreated (−) cells visualized by SDS-PAGE with Far-Red excitation and 715/30 filter emission (white bands) and Stain-Free imaging (black bands) show a band at approximately 61.5 kDa in the Halo-N-EPG (+) group that is not present in the untreated (−) group—indicative of the presence of a GPI anchor. Halo-C-EPG did not present any JFX650 associated bands. The positive control, Halo-N-CD59, presented bands at approximately 51.5 kDa. Mock transfected cells did not present any JFX650 associated bands.

EPG is structurally very similar to members of the Ly6/uPAR family. These proteins are characterized by the presence of a three-finger Ly6/uPAR structural domain formed by disulfide bonds between cysteine residues [16]. A significant portion of proteins in this family are associated with the membrane via GPI anchor—including the human protein CD59 [16]. The idea that EPG may be GPI anchored came to light after observing that Halo-C-EPG does not localize to the membrane as indicated in figure 1. One possibility is that in this construct, HaloTag is blocking a critical signalling domain on the C terminal end of EPG. This putative signalling domain remains consistent with the way that GPI anchored proteins are formed—translation begins at the N terminus and concludes with the C terminal GPI anchoring sequence being cleaved off and replaced with a GPI anchor in the endoplasmic reticulum [17]. Additionally, lysate of HeLa cells expressing Halo-C-EPG and Halo-N-EPG present very differently when run on an SDS-PAGE gel (figure 2*a*). Halo-C-EPG presents exactly as expected—46 kDa—equivalent to the molecular weight of EPG and HaloTag combined. Halo-N-EPG, however, presents as a series of bands at a higher molecular weight (between 50 and 75 kDa), indicating some sort of posttranslational modification of EPG. The series of bands could be due to several different types of posttranslational modifications, or possibly immature versions of the protein that have not yet received proper modifiers. Halo-N-EPG and Halo-C-EPG present in this same manner in several different cell types as shown in electronic supplementary material, figure S1, indicating that specialized machinery is not necessary for expression of either construct. The known GPI anchored protein CD59, when expressed in HeLa cells as Halo-N-CD59, also presents at a higher molecular weight (approx. 51 kDa versus the expected 46 kDa) as shown in figure 2*a*. The increase in molecular weight specifically points to the GPI anchor which has been previously described to bind high quantities of SDS effectively ‘increasing’ the molecular weight of GPI anchored proteins [18]. Other fluorescent bands present in the gel are likely HaloTag that is no longer fused to the protein of interest (approx. 35 kDa). As a control to ensure HaloTag does not non-specifically bind to other proteins native to HeLa cells, a mock transfected group was subject to the same labelling, lysis and SDS-PAGE which yields no JFX650 fluorescent bands.

To test whether EPG is GPI anchored, we used the enzyme phosphatidylinositol specific phospholipase C (PI-PLC). This enzyme specifically cleaves the phosphatidylinositol of the GPI anchor, and effectively releases any GPI anchored protein into the external cellular environment as illustrated in figure 2*b*. HeLa cells expressing various constructs were labelled with JFX650 and treated with PI-PLC (+); control groups were subject to the same treatment without the addition of the PI-PLC (−). After treatment, medium was carefully collected off of the top of the cells and run on an SDS-PAGE gel. By using Far-Red excitation paired with a 715/30 emission filter, we can specifically visualize HaloTag-EPG fusion constructs within the gel due to the JFX650 ligand. As shown in figure 2*c*, we observe a band at 61.5 kDa from Halo-N-EPG expressing cells that is not present in its untreated counterpart. This result indicates that Halo-N-EPG is GPI anchored. Halo-C-EPG does not exhibit fluorescent bands in either group as expected due to its localization. Halo-N-CD59 also exhibits a clear band in the PI-PLC treated group; this band is also observed in the untreated group, but at a much lesser quantity. These results act as a positive control ensuring the assay is valid as CD59 is known to be GPI anchored. Mock transfected HeLa cells that were labelled and subjected to the same PI-PLC treatments do not exhibit bands in either group. As demonstrated with the mock transfected groups, and in electronic supplementary material, figure S2, JFX650 is highly specific to HaloTag and does not bind non-specifically to native proteins in HeLa cells or other cell lines.

### 2.3. Analysis of EPG’s localization and function when HaloTag is fused to each terminus

**Figure 3.**
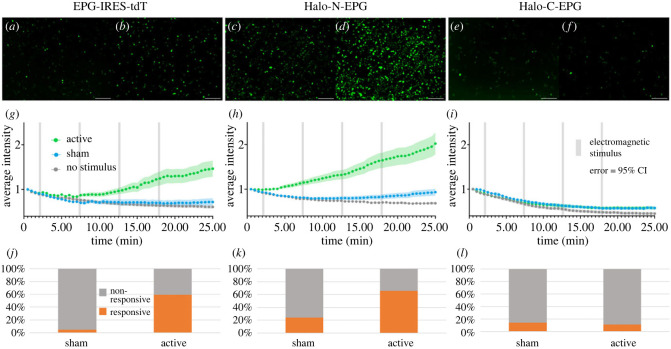
Developing an assay using GCaMP6m to determine if EPG is still functional after the addition of HaloTag. (*a–f*) HeLa cells expressing GCaMP6m and various EPG-HaloTag fusion constructs visualized before (*a,c,e*) and after (*b,d,f*) electromagnetic stimulation. (*a*,*b*) Cells expressing EPG-IRES-tdT appear more intense after stimulation. (*c*,*d*) Cells expressing Halo-N-EPG appear more intense after stimulation. (*e*,*f*) Cells expressing Halo-C-EPG appear relatively unchanged after stimulation. Scale bars represent 200 μm. (*g–i*) Average intensity of GCaMP6m over time with various electromagnetic stimuli. Error bars are representative of 95% CI. Significant increases in intensity were observed between the no stimulus/sham and active groups in both (*g*) (*p* < 0.0001, unpaired *t* test) and (*h*) (*p* < 0.0001, unpaired *t* test). No significant difference was observed between any groups in (*i*). (*j–l*) Percentage of individual cells that produced a signal greater than 3×SD+mean of the corresponding no stimulus group. Significant differences were observed between sham and active groups in both (*j*) and (*k*). No significant difference was observed between sham and active groups in (*l*). The EPG-IRES-tdT group included *n* = 177, *n* = 134, and *n* = 163 cells over four experiments for no stimulus, sham, and active groups respectively. The Halo-N-EPG group included *n* = 282, *n* = 269, and *n* = 279 cells over four experiments for no stimulus, sham, and active groups respectively. The Halo-C-EPG group included *n* = 160, *n* = 182, and *n* = 189 cells over three experiments for the no stimulus, sham, and active groups respectively.

Previous reports indicate that upon magnetic stimulation, EPG causes an increase in intracellular calcium when expressed in mammalian cells [11]. We chose to build on this principle, creating a functional assay for EPG. The assay relies on GCaMP6m as a sensor for intracellular calcium levels [9]. In theory, mammalian cells co-transfected with EPG and GCaMP6m will experience an increase in cytosolic calcium upon magnetic stimulation. This increase in cytosolic calcium ions will be reflected by an increase in fluorescence of the calcium reporter GCaMP6m. This idea was confirmed using an EPG-IRES-tdTomato (tdT) construct (figure 3) where expression and visualization of the fluorescent tdT confirm expression of EPG. HeLa cells were grown in 35 mm tissue culture dishes and stimulated with a custom electromagnetic air-core coil that fits a 35 mm dish in its centre [20]. The coil utilizes double wrapped copper wires that allow for both active and sham stimuli to be produced. The active stimulus is produced when current is run in the same direction in both wires. The sham stimulus is produced when current is run in opposite directions; this anti-parallel configuration cancels the formation of a magnetic field [20]. The sham is an ideal control in this case because the sample is still subjected to the heat and electricity associated with the magnet, without the magnetic field [5,21].

Cells were stimulated using a pulse pattern and fluorescence of GCaMP6m was observed for the duration of the experiment using a GFP filter (Keyence BZ-X) to yield a video such as video S1 in the electronic supplementary material. The pulse pattern was chosen to avoid overheating of the electromagnetic coil that would ensue if it were used to produce a constant stimulus at a high voltage. Data for co-transfected cells (determined by overlaying fluorescent images as shown in electronic supplementary material, figure S3) were gathered by placing regions of interest (ROIs) using the Time Series Analyzer V3 package [22] for FIJI [23]. The intensity values for each ROI were normalized to the first point in the read—allowing for clearer visual representation of the data. Intensities were then averaged between all ROIs over four experiments to produce the graph shown in figure 3*g*. Cells that received the active stimulus have a higher average intensity than cells that received the sham stimulus and cells that received no stimulus.

This functional assay was then applied to the HaloTag-EPG fusion proteins to evaluate if HaloTag altered the function of EPG. Figure 3*h* shows that Halo-N-EPG maintained the native function of EPG upon electromagnetic stimulation demonstrated by the increase in average intensity observed in the active group. Figure 3*i* shows that Halo-C-EPG does not maintain its function as no increase in intensity was observed. Figure 3*j*,*k* represents data for individual ROIs compared to a threshold of 3×SD+mean of their corresponding no stimulus group. ROIs above that threshold were considered ‘responsive’ and cells below the threshold were considered ‘non-responsive’. The EPG-IRES-tdT constructs yielded 59% ‘responsive’ which remains relatively consistent with the Halo-N-EPG groups that yielded 66% ‘responsive’. The Halo-C-EPG groups yielded 11% ‘responsive’ that may be attributed to background cell function.

### 2.4. Observing how homologues of EPG from different species respond to electromagnetic stimulation

**Figure 4.**
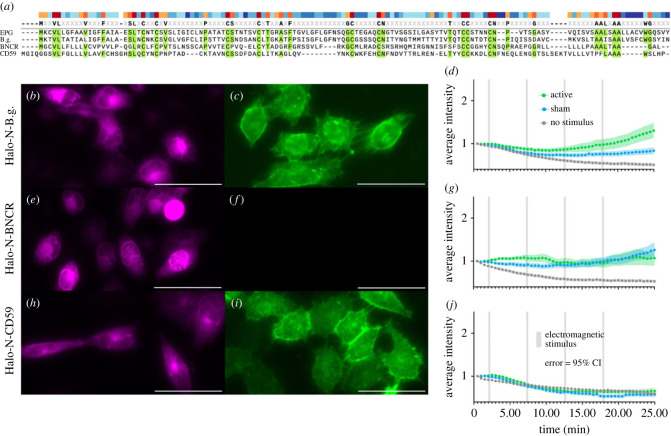
Observing the effect of electromagnetic stimulation on homologues of EPG from different species. (*a*) Alignment of amino acid sequences for EPG and homologues B.g., BNCR, and CD59. (*b,c*) HeLa cells expressing Halo-N-B.g. and labelled with AF488 (*b*) and JFX650 (*c*) demonstrating this protein is membrane-associated. (*d*) Average intensity of GCaMP6m in HeLa cells expressing Halo-N-B.g. with various stimuli. (*e*,*f*) HeLa cells expressing Halo-N-BNCR and labelled with AF488 (*e*) and JFX650 (*f*) showing the protein, unexpectedly, does not localize to the membrane. (*g*) Average intensity of GCaMP6m in HeLa cells also expressing Halo-N-BNCR with various stimuli. (*h*,*i*) HeLa cells expressing Halo-N-CD59 labelled with AF488 (*h*) and JFX650 (*i*) demonstrating this protein is membrane-associated. (*j*) Average intensity of GCaMP6m in HeLa cells also expressing Halo-N-CD59 with various stimuli. (*d,g,j*) Error bars are representative of 95% CI. All scale bars indicate 50 μm. The Halo-N-B.g. group included *n* = 84, *n* = 97, and *n* = 91 cells over 3 experiments for no stimulus, sham, and active groups respectively. The Halo-N-BNCR group included *n* = 118, *n* = 120, and *n* = 105 cells over 3 experiments for no stimulus, sham, and active groups respectively. The Halo-N-CD59 group included *n* = 130, *n* = 124, and *n* = 120 cells over 4 experiments for the no stimulus, sham, and active groups respectively.

To examine the uniqueness of EPG's function, we chose to observe how homologues from a range of species would respond to electromagnetic stimulation using the same functional assay. One ideal comparison is a homologous protein from the bluntnose knifefish *Brachyhypopomus gauderio*—a species of electric fish. The protein was identified in the transcriptome of the B.g. fish but remains unnamed and uncharacterized; therefore we will refer to it as ‘B.g.’ [24]. Another ideal homologous control is the protein Bouncer (BNCR) that comes from the zebrafish *Danio rerio*—this species represents a fairly well-studied species of non-electric fish [25]. Lastly, we revisit the homologous human protein, CD59. Homology between EPG and the three control proteins is shown in figure 4*a*—conserved amino acids are highlighted in green and similarity of amino acids is indicated by the colour of the bars on top.

B.g. is a similar size to EPG and likely adopts a similar structure to the Ly6/uPAR family determined by the conserved cysteine residues. Labelling Halo-N-B.g. with permeable JFX650 (figure 4*b*) and impermeable AF488 (figure 4*c*) indicates this protein is adequately expressed in HeLa cells and is associated with the membrane. When subject to the functional assay as described above, B.g. seems to have some response to both the active and sham stimuli (figure 4*d*). Due to the electromagnetic coil design, an electrical field will be present as the voltage coming from the power supply ramps up to and down from the desired voltage. This period lasts about one second at the beginning and one second at the end of each of the four pulses [20]. The observed responses may be attributed to the protein coming from a species of electric fish; evolutionarily it may be possible that the ability to sense electromagnetic fields emerged from the ability to sense electric fields or vice versa. Further indication of a relationship between magnetoreception and electroreception is that EPG was obtained from the ampullary organs of the glass catfish [11]. Ampullae of Lorenzini are typically found in cartilaginous fish species, and are known to be responsible for electroreception in certain species [26]. Future studies may find it worthwhile to further examine the evolutionary relationship between the glass catfish, and species of electric fish such as *Brachyhypopomus gauderio* to examine this idea.

BNCR is known to adopt the Ly6/uPAR structural domain and is membrane associated—hypothetically via GPI anchor [25]. Labelling Halo-N-BNCR with permeable JFX650 (figure 4*e*) and impermeable AF488 (figure 4*f*) indicates this particular construct is not associated with the membrane despite being adequately expressed in HeLa cells. In this instance, HaloTag likely interfered with the native structure or function of BNCR. When subjected to the functional assay, BNCR appears to have exhibited a minimal response to the sham and active stimuli equally. Because the native localization of BNCR was not maintained, these results do not accurately reflect its physiology; the results do however serve as an interesting point of comparison that may influence further characterization of BNCR.

CD59 is another member of the Ly6/uPAR family and is ubiquitously expressed in human tissue. It is a GPI anchored protein known to act as an inhibitor of the formation of the membrane attack complex (MAC) [27]. Labelling Halo-N-CD59 with permeable JFX650 (figure 4*h*) and impermeable AF488 (figure 4*i*) indicates that Halo-N-CD59 is membrane-associated and is adequately expressed in HeLa cells. When subject to the functional assay, there was no response to any stimuli as shown in figure 4*j*. To make comparison of groups easier, electronic supplementary material, figure S4, shows all functional assay graphs side-by-side. Electronic supplementary material, figure S5, also includes data representing the percentage of individual cells that responded in each group displayed side-by-side for comparison. Together, these results indicate that EPG has a unique response to electromagnetic stimulation, but leave questions regarding the evolution of magnetoreception and electroreception in fish. These results also solidify the use of CD59 as a useful control against EPG in these, and future, experiments.

### 2.5. A phenylalanine rich region in EPG is critical for its functionality

**Figure 5.**
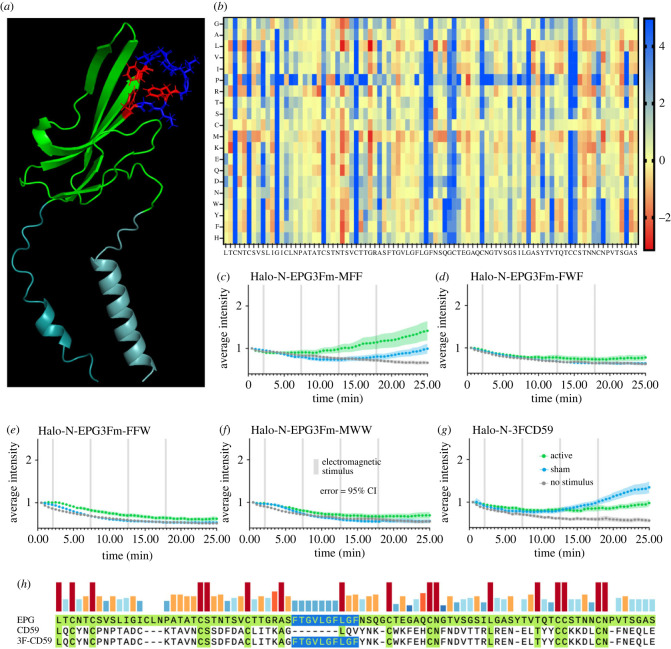
Site directed mutagenesis of the three-phenylalanine region results in change of function. (*a*) Predicted structure of EPG with the three phenylalanine residues highlighted in red. (*b*) Stability of amino acid swaps for EPG in a heatmap; red is a stabilizing swap, yellow is neutral, and blue is a destabilizing swap. (*c–g*) Average intensity of GCaMP6m in HeLa cells expressing various 3F mutants over time with various stimuli. Error bars are representative of 95% CI. (*c–f*) Amino acids were swapped with the most stabilizing residue indicated in (*b*). (*c*) First F in 3F region knocked out. (*d*) Second F in 3F region knocked out. (*e*) Third F in 3F region knocked out. (*f*) All three F residues in 3F region knocked out. (*g*) 3F motif inserted into CD59. The Halo-N-EPG3Fm-MFF group included *n* = 77, *n* = 90, and *n* = 89 cells over 3 experiments for no stimulus, sham, and active groups respectively. The Halo-N-EPG3Fm-FWF group included *n* = 87, *n* = 96, and *n* = 86 cells over 3 experiments for the no stimulus, sham, and active groups respectively. The Halo-N-EPG3Fm-FFW group included *n* = 87, *n* = 85, and *n* = 88 cells over 3 experiments for the no stimulus, sham, and active groups respectively. The Halo-N-EPG3Fm-MWW group included *n* = 84, *n* = 83, and *n* = 89 cells over 3 experiments for the no stimulus, sham, and active groups respectively. The Halo-N-3FCD59 group included *n* = 86, *n* = 92, and *n* = 95 cells over 3 experiments for no stimulus, sham, and active groups respectively.

To determine how EPG senses and responds to magnetic stimulation, we elected to look closer at its structure in comparison to several homologues from the Ly6/uPAR family. One notable region that stood out is the ‘3F region’ named for being rich in phenylalanine residues. These phenylalanine residues are relatively conserved between EPG and homologues from species of electric fish, but not homologues from humans or other mammalian species (electronic supplementary material, figure S6). Figure 5*a* indicates the position of the three phenylalanine residues in the 3F region in the predicted structure of EPG. The positioning of the aromatic side chains may allow for pi-stacking or facilitate holding a charge. To determine if this region is involved with EPG’s function, we knocked out each of the three phenylalanine residues individually and consecutively with the most stabilizing swap determined by ΔΔG calculations which are visually represented in the heatmap shown in figure 5*b*. The phenylalanine at position 55 was swapped for methionine (F55M) using site directed mutagenesis and subjected to the functional assay described above. As shown in figure 5*c* the response is relatively consistent with that of native EPG, although slightly diminished. This indicates F55 is not critical for functionality, but aids in its efficacy. The phenylalanine at position 61 was swapped in the same manner for tryptophan (F61W), and its response was also gauged using the functional assay. Figure 5*d* demonstrates a loss of function after this mutation. The phenylalanine at position 64 was swapped for tryptophan (F64W) and is demonstrated in figure 5*e* to also cause a loss of function. Finally, all three F residues were swapped simultaneously (F55M, F61W, F64W) and this mutant was tested using the functional assay. Again, we observe a loss of function as shown in figure 5*f*. These data demonstrate that F61 and F64 are critical for functionality, and that the 3F motif as a whole is essential for magnetoreception.

In addition to mutating EPG, we also sought to insert the 3F region into CD59 to determine whether we could alter the function in a homologue that previously showed no response. Once again using site directed mutagenesis, the 3F region was inserted into CD59 in a way that conserved the critical positioning of the cysteine residues. Figure 5*h* shows the 3F motif highlighted in blue that was taken from EPG and inserted into CD59 to form 3FCD59. When subject to the functional assay (figure 5*g*), 3FCD59 exhibits different functionality from that of CD59, further indicating the 3F motif may be a critical factor for sensing EMF.

## 3. Conclusion

Overall, these findings provide new insight into the structure and function of EPG. This information may serve as a foundation for future work involved with understanding and utilizing the magnetoreceptive abilities of EPG. This work also represents an important step to understanding magnetoreception in biological systems as a whole by providing an example of how a magnetoreceptive protein may function and what its structure may look like. While this study was able to conclude many specifics pertaining to EPG, many questions remain unanswered. Future research may build upon this study by determining the specific pathway EPG takes part in to influence calcium concentrations, or by determining other aspects of EPG's structure that are critical to its function.

## 4. Material and methods

### 4.1. Experimental model and subject details

All cell lines were maintained in 25 cm^2^ polystyrene flasks stored in a humidified incubator at 37°C with 5% CO_2_. HeLa, HEK-293, and MDA-231 cells were cultured in DMEM (ThermoFisher) supplemented with 10% FBS (ThermoFisher) and 1% PenStrep (ThermoFisher). 9L/LacZ cells were maintained in DMEM (ThermoFisher) supplemented with 10% FBS (ThermoFisher). RIN-5F cells were cultured in RPMI 1640 medium (ThermoFisher) supplemented with 10% FBS (ThermoFisher) and 1% PenStrep (ThermoFisher).

### 4.2. Plasmid construction and site directed mutagenesis

Primers and g-blocks used to generate constructs for this study were ordered from IDT. The GCaMP6m plasmid was obtained from Addgene [19]. EPG-IRES-tdT was previously synthesized in the laboratory for use in other projects. Halo-N-EPG was constructed so that the N-terminal signal sequence of EPG preceded the HaloTag sequence followed by the rest of EPG. Halo-C-EPG contains the entirety of EPG followed by the entirety of the HaloTag sequence. All other Halo-N constructs (i.e. BNCR, CD59, B.g.) were cloned by removing the section of EPG following HaloTag and substituting in the gene of interest. EPG's signal sequence remained preceding HaloTag in all the constructs to increase the likelihood that the proteins would make it to the membrane. All cloning was completed with the NEBuilder HiFi DNA Assembly kit (NEB). To generate the mutant constructs (i.e. Halo-N-3FCD59 and Halo-N-EPG3FK constructs), primers with the desired mutations were used to conduct site directed mutagenesis via PCR.

### 4.3. GCaMP6m functional assay

HeLa cells were plated in 35 mm tissue culture dishes (Falcon) at a density of approximately 0.1 × 10^6^ cells and allowed to grow for 24 h. Cells were transfected with both the construct of interest and GCaMP6m using the Lipofectamine 3000 kit (Invitrogen) according to the manufacturer instructions. Cells were labelled with 200 nM JFX650 HaloTag ligand (Janelia Materials) 24 h post-transfection. The ligand was allowed to bind for 15 min, the medium was removed followed by two washes with PBS (Corning) to remove excess ligand, and the cells were covered with prewarmed media. Cells were imaged in a BZ-X770 Keyence microscope using a 10× objective while maintained in a Tokai-Hit chamber at 37°C with 5% CO_2_ and humidity. Cells were visualized with either the TritC filter (Keyence BZ-X) to identify tdT or the Cy5 filter (Keyence BZ-X) to identify cells labelled with JFX650—both indicative of successful expression of the construct of interest. Cells were visualized with the GFP filter (Keyence BZ-X) to show GCaMP6m over 25 min. Control groups remained undisturbed for the duration of the experiment. Active and sham groups were stimulated with a custom air-core electromagnetic coil [20] at 4.5 A (14.5 mT active; 0.3 mT sham) for 15 s followed by 5 min of rest for 4 pulses for the duration of the experiment. Cells were grown, transfected and imaged in groups of three so that one group of cells received no stimulus, one group of cells received sham stimulus, and one group received active stimulus.

### 4.4. Membrane localization imaging

HeLa cells were plated in 96-well glass-bottom plates (Costar) and allowed to grow for 24 h prior to transfection. Cells were transfected with the construct of interest using the Lipofectamine 3000 kit (ThermoFisher) according to the manufacturer instructions. Half of the wells were labelled with JFX650 (Janelia Materials) and the other half with AF488 (Promega). HaloTag ligands were allowed to incubate with the cells for 15 min; excess ligand was then removed by aspirating the media and washing twice with PBS. The cells were covered in prewarmed Fluorobrite DMEM (ThermoFisher) and visualized using a BioTek Cytation 5 Imaging Reader and a 40× objective. AF488 was viewed with the GFP filter (Agilent) and JFX650 was visualized with the Cy5 filter (Agilent).

### 4.5. Lysate analysis

HeLa cells were plated in 6-well plates (Corning) at a density of approximately 0.1 × 10^6^ cells per well and allowed to grow for 24 h. One well was transfected with each construct, Halo-N-EPG, Halo-C-EPG, Halo-N-CD59, and sterile ddiH_2_O (mock) using the Lipofectamine 3000 kit according to the manufacturer instructions. The cells were allowed to grow for 24 h and were then labelled with 200 nM JFX650 (Janelia Materials) and allowed to incubate for 15 min. Excess HaloTag ligand was removed by aspirating the media and washing twice with PBS. Each well was lysed in 100 µl of 1× Laemmli sample buffer (Sigma Aldrich) and mechanically separated from the bottom of the plate. The cell lysates were boiled for 5 min at 95°C, then 10 µl of each was loaded into a Stain-Free Any kD Mini PROTEAN SDS-PAGE gel (Bio-Rad) and run at 200 V for approximately 40 min. The gel was visualized using a Chemi-Doc MP Imaging System (Bio-Rad) with Far-Red excitation and 715/30 filter emission to show only JFX650 labelled products. The gel was then visualized with Bio-Rad Stain-Free imaging to show non-specific products as a method of loading control. The images were then overlayed and analysed using the Bio-Rad Image Lab Software.

### 4.6. Phosphatidylinositol-specific phospholipase C assay

HeLa cells were plated in 6-well plates (Costar) at a density of approximately 0.05 × 10^6^ cells per well. Prior to seeding cells, plates were treated with Poly-D-Lysine (ThermoFisher) to ensure the cells did not fall off the plate during the experiment. Cells were transfected with either Halo-N-EPG, Halo-C-EPG, Halo-N-CD59, or sterile ddiH_2_O (mock) using the Lipofectamine 3000 kit (Invitrogen) according to the manufacturer instructions. After 24 h the cells were labelled with 200 nM JFX650 (Janelia Materials) and allowed to incubate for 15 min. Excess HaloTag ligand was removed by aspirating the media and washing twice with PBS. One well of cells expressing each construct was treated with 0.25 units of PI-PLC (ThermoFisher) in 250 µl of cold PBS; other wells expressing each construct were left untreated and only covered in cold PBS. Plates were rocked at 4°C for 20 min and the buffer was carefully collected off the top of the cells for analysis. 10 µl of each collected buffer was mixed with 2× Laemmli sample buffer and loaded into a Stain-Free Any kD Mini PROTEAN SDS-PAGE gel (Bio-Rad). The gel was run at 200 V for approximately 40 min. The gel was then visualized using a Chemi-Doc MP Imaging System (Bio-Rad) with Far-Red excitation and 715/30 filter emission to show only JFX650 labelled products. The gel was then visualized with Bio-Rad Stain-Free imaging to show non-specific products as a method of loading control. The images were then overlayed and analysed using the Bio-Rad Image Lab Software.

### 4.7. Protein structure analysis

The structure of EPG was predicted using RoseTTAFold [28] protein structure prediction software and visualized with the PyMol molecular visualization system. Amino acid alignments of EPG and homologues were generated using SnapGene. ΔΔG calculations were completed with FoldX.

### 4.8. GCaMP6m functional assay data analysis

Twenty-five-minute videos of cells were split into 50 images (one image every 30 s) for analysis. The Time Series Analyzer V3 [22] was used in conjunction with FIJI [23] to place ROIs around viable cells that were confirmed to be co-transfected (i.e. tdT or JFX650 fluorescence and GCaMP6m fluorescence as demonstrated in electronic supplementary material, figure S3). The ROI was placed so that the cell remained within the borders in all 50 frames with minimal background inclusion. Intensity values for each ROI at each time point were gathered, then normalized to the first point in the read such that each ROI had a starting intensity of one. Intensity values for every ROI over every experiment were averaged and plotted using PRISM 10 (GraphPad) to create graphs showing the average intensity over time. The error bars displayed represent a 95% CI. Unpaired *t* tests were conducted using PRISM 10 to determine significant differences between groups.

### 4.9. GCaMP6m functional assay individual cell analysis

Intensity over time for each individual cell/ROI was plotted and compared to a threshold of 3×SD+mean of the corresponding no stimulus group. Cells were considered ‘responsive’ if they had an intensity greater than the threshold, or ‘non-responsive’ if they had an intensity less than the threshold. Cells were excluded if they oscillated above and below the threshold or exhibited anomalies such as a single spike above the threshold. The total number of ‘responsive’ and ‘non-responsive’ cells was totalled for every experiment to generate percentages shown as bar graphs.

## Data Availability

DNA sequences for all constructs utilized in this paper can be found in the associated electronic supplementary material [29].
